# Molecular Detection of *Bartonella henselae* in Healthy Cats from Portugal (2015–2025): One Health Context and Implications for Transfusion Medicine

**DOI:** 10.3390/pathogens15020131

**Published:** 2026-01-26

**Authors:** Ricardo Lopes, Hugo Lima de Carvalho, Filipe Sampaio, Cátia Fernandes, Cristina Costa Santos, Carlos Sousa, Ana Rita Silva, Rita de Sousa, Hugo Silva, Ana Patrícia Lopes, Elsa Leclerc Duarte, Luís Cardoso, Ana Cláudia Coelho

**Affiliations:** 1 Department of Veterinary Sciences, University of Trás-os-Montes e Alto Douro (UTAD), 5000-801 Vila Real, Portugal; lopes.rmv@gmail.com (R.L.); aplopes@utad.pt (A.P.L.); accoelho@utad.pt (A.C.C.); 2 Department of Veterinary and Animal Sciences, University Institute of Health Sciences (IUCS), CESPU, 4585-116 Gandra, Portugal; 3 CEDIVET Veterinary Laboratories, Lionesa Business Hub, R. Lionesa 446 C24, 4465-671 Leça do Balio, Portugal; hugo.carvalho@cedivet.pt (H.L.d.C.); filipe.sampaio@cedivet.pt (F.S.); 4 Molecular Diagnostics Laboratory, Unilabs Portugal, Lionesa Business Hub, R. Lionesa, 4465-671 Leça do Balio, Portugal; hugo.silva@unilabs.com; 5 Cytology and Hematology Diagnostic Services, Laboratory of Histology and Embryology, Department of Microscopy, ICBAS-School of Medicine and Biomedical Sciences, University of Porto (U. Porto), Rua de Jorge Viterbo Ferreira, 228, 4050-313 Porto, Portugal; 6 AniCura Santa Marinha Veterinary Hospital, R. Dom Henrique de Cernache 183, 4400-625 Vila Nova de Gaia, Portugal; catia.fernandes@anicura.pt; 7 RISE-Health, Department of Community Medicine, Information and Health Decision Sciences, Faculty of Medicine, University of Porto (U. Porto), Alameda Prof. Hernâni Monteiro, 4200-319 Porto, Portugal; csantos@med.up.pt; 8 Molecular Pathology Laboratory, SYNLAB Portugal, Av. Columbano Bordalo Pinheiro, 75A, 1070-061 Lisboa, Portugal; carlos.sousa@synlab.pt (C.S.); ana-rita.silva@synlab.pt (A.R.S.); 9 PerMed Research Group, RISE-Health, Faculty of Medicine, University of Porto (U. Porto), 4200-319 Porto, Portugal; 10 Centre of Study of Vectors and Infectious Diseases, National Institute of Health Dr. Ricardo Jorge, Av. da Liberdade 5, 2965-575 Águas de Moura, Portugal; rita.sousa@insa.min-saude.pt; 11 Animal and Veterinary Research Centre (CECAV), Associate Laboratory for Animal and Veterinary Sciences (AL4AnimalS), University of Trás-os-Montes e Alto Douro (UTAD), 5000-801 Vila Real, Portugal; 12 Department of Veterinary Medicine, School of Science and Technology, University of Évora, Polo da Mitra, Apartado 94, 7002-554 Évora, Portugal; emld@uevora.pt; 13 Mediterranean Institute for Agriculture, Environment and Development (MED), Global Change and Sustainability Institute (CHANGE), University of Évora, Polo da Mitra, Apartado 94, 7002-554 Évora, Portugal

**Keywords:** One Health, qPCR, Sanger sequencing, transfusion-transmissible infection, vector-borne infection

## Abstract

*Bartonella henselae* is a flea-borne zoonotic bacterium for which domestic cats constitute the principal reservoir. However, contemporary molecular epidemiological data from Portugal remain scarce. This retrospective laboratory study analysed EDTA-stabilised blood samples from apparently healthy cats submitted for routine screening by 74 veterinary centres across mainland Portugal and autonomous regions over an 11-year period (2015–2025). DNA extracts were tested using a species-specific TaqMan qPCR assay for *B. henselae* with an internal extraction control, and a subset of samples was subsequently confirmed by nested PCR followed by Sanger sequencing (*ribC*). Among 270 cats, 47 tested positive, yielding a qPCR prevalence of 17.4% (95% confidence interval [CI] 13.1–22.5). Submissions were predominantly from Northern Portugal, and infection status was not statistically associated with the Nomenclature of Territorial Units for Statistics (NUTS) level 2 region (*p* = 0.478). Infection was more frequent in younger cats (median age 2 years, interquartile range [IQR] 1–5; *p* = 0.037), while sex (*p* = 0.103) and breed (*p* = 0.730) were not significantly associated with infection status. These findings support endemic circulation of *B. henselae* in Portuguese cats at levels comparable to other temperate European regions. The detection of subclinical infection in apparently healthy cats is relevant to transfusion medicine and supports the inclusion of *B. henselae* qPCR screening in donor selection protocols.

## 1. Introduction

The genus *Bartonella* comprises fastidious, Gram-negative, facultative intracellular alphaproteobacteria with a distinctive haemotropic tropism, colonising mammalian erythrocytes and endothelial cells, and most commonly transmitted by arthropod vectors [1,2,3,4,5,6,7,8,9,10,11,12,13,14,15,16,17,18]. These organisms have a worldwide distribution and are considered important emerging zoonotic agents in both veterinary and human medicine, with more than 45 species now recognised with additional taxa continuing to be described [2,3,19,20,21,22,23,24,25,26]. Among these, *Bartonella henselae* and related species such as *B. clarridgeiae* are recognised as the most important from both a veterinary and public health perspective, serving as the primary aetiological agent of cat-scratch disease (CSD) in humans [12,17,20,27,28,29]. Domestic and stray cats (*Felis catus*) represent the principal natural reservoir for *B. henselae*, typically maintaining a persistent and subclinical bacteraemia that facilitates the pathogen’s dispersal [1,30]. Beyond bloodstream infection, *Bartonella* spp. DNA has been detected in feline reproductive/placental tissues, but transplacental transmission remains unproven [31].

The transmission of *B. henselae* within feline populations is primarily mediated by the cat flea *Ctenocephalides felis* through the inoculation of infected flea faeces into the skin via scratches or bites [1,32,33]. Epidemiologically, *B. henselae* shows marked geographic heterogeneity, with higher prevalence of feline infection reported in climatic settings that favour year-round flea survival and sustained vector pressure [21,22,23]. In North America, this pattern is reflected by substantially higher cat seroprevalence in warm, humid regions (approx. 34–55%) compared with cold and/or arid areas (approx. 4–7%) [34]. Consistently, global meta-analytic evidence indicates increased feline *Bartonella* spp. prevalence within warmer latitudinal belts [35]. Across studies, the overall molecular prevalence of *Bartonella* spp. in cats is estimated at ~15%, with *B. henselae* predominating (13.1%) and comparatively lower detection of *B. clarridgeiae* (1.7%) and *B. koehlerae* (0.1%) [35]. In Western Europe, pooled prevalence is ~19% [35], whereas Portuguese studies report variable PCR positivity, ranging from 2.9% in Southern Portugal with *B. henselae* and *B. clarridgeiae* confirmed [30] at 20.3% in stray cats in Lisbon, as determined by quantitative PCR [1].

In humans, CSD and related clinical syndromes have been documented in all populated continents and reported seroprevalence ranges from <1% to >40%, largely reflecting differences in exposure intensity (e.g., frequency of cat contact), occupational risk, and rural–urban context [22,23,36,37,38,39,40,41]. Although *B. henselae* is widely distributed in domestic cats, confirmed human cases in Portugal appear infrequent and likely under-recognised. Most molecular and serological investigations have been negative, including a 2022 study [1] at the Institute of Hygiene and Tropical Medicine (IHMT) that detected no *Bartonella* spp. DNA in 30 healthy, cat-exposed individuals, while data from the National Institute of Health (INSA) reports a 17% seroprevalence among 189 suspected human cases [42]. The absence of systematic surveillance and the fact that CSD is not a notifiable disease contribute to substantial under-recognition of its true incidence and public health impact [1,42,43].

Risk factors identified for feline *Bartonella* spp. infection in Portugal are consistent with those reported internationally, with infection being associated with younger age (particularly <2 years), outdoor access or free-roaming behaviour, flea infestation, and the absence of effective ectoparasite control [30,44,45,46,47]. In the survey in Southern Portugal [30], PCR positivity was concentrated in strays or outdoor-access cats without regular flea prevention, supporting the importance of vector exposure. Higher-density environments such as colonies, shelters, and multi-cat households also appear to increase risk, likely by facilitating flea exchange and inter-cat transmission [30,44,45,47]. In contrast, associations with sex or breed have not been consistently demonstrated across studies [44,45,46,48,49,50].

The diagnosis of *Bartonella* spp. infection is influenced by assay-dependent limitations that can bias epidemiological inference. In cats, infection is frequently occult and characterised by chronic, relapsing, intermittent bacteraemia with low-grade and fluctuating bacterial loads, contributing to frequent culture-negative results and reduced serological specificity due to cross-reactivity [27,51,52]. Blood PCR is, therefore, most informative during periods of active bacteraemia, but its sensitivity is suboptimal in low-burden or intermittent infection and may be further compromised by prior antimicrobial exposure and pre-analytical variables [44,47,53]. Although culture remains the diagnostic gold standard, it is technically demanding and often requires prolonged incubation, which limits routine implementation [29,54,55,56,57]. Serology is more frequently positive than PCR, consistent with previous exposure and/or latent infections, but it does not confirm active infection [27,51,52,58]. Modern methodologies (e.g., *nuoG* qPCR, enrichment culture such as *Bartonella* Alpha-Proteobacteria Growth Medium (BAPGM), and multilocus sequencing) substantially enhance detection, but are not widely available [1,35,51,59,60,61,62]. Consequently, the true prevalence in Portuguese cats and humans is likely underestimated. A multimodal strategy integrating serology and PCR, with culture when feasible, is therefore recommended, and culture pre-enrichment followed by PCR may improve sensitivity in low-level or intermittent bacteraemia [44,46,47,53].

From a One Health standpoint, Portugal seems to resemble other Southern European regions in which *B. henselae* circulates in feline populations, overt disease in cats is uncommon, and human CSD and endovascular infection remain sporadic. Nonetheless, the potential for severe morbidity, particularly in immunocompromised individuals, and evidence of heightened exposure among veterinarians and cat rescue workers in other regions support proportionate preventive measures, including rigorous flea control in cats, education to minimise bites and scratches, and targeted diagnostic evaluation in compatible human presentations [1,20,27,35,52,63,64,65,66].

This study aimed to characterise the molecular epidemiology of *B. henselae* infection in apparently healthy cats in Portugal, using a retrospective qPCR dataset (2015–2025), estimating the prevalence of qPCR-positive submissions, describing spatiotemporal patterns of positivity with a focus on geographical heterogeneity at the Nomenclature of Territorial Units for Statistics [NUTS] level 2 region, and assessing associations between PCR status and host variables (age, sex and breed), while contextualising the findings within a One Health framework, with attention given to implications for zoonotic risk awareness and evidence-informed transfusion medicine strategies, including donor-cat screening and effective ectoparasite control.

## 2. Materials and Methods

### 2.1. Sampling, Data Collection and Diagnostic Procedures

Peripheral EDTA-anticoagulated whole-blood samples from apparently healthy cats submitted for routine preventive screening were submitted to CEDIVET Veterinary Laboratories (Portugal) by 74 veterinary medical centres, including first-opinion practices and referral hospitals, across mainland Portugal and the autonomous regions. For each submission, a standard laboratory requisition provided anonymised signalment data, namely age, sex and breed. Cases were included if breed and sex were recorded. Age data were missing in 25.9% of submissions. These cases were retained for overall prevalence estimation but excluded from age-specific analyses. Age was categorised a priori into five groups: kitten (<1 year), young (1 to <2 years), adult (2 to <6 years), senior (6 to <11 years) and geriatric (≥11 years) [67,68,69,70,71,72].

### 2.2. Molecular Analysis

For each sample, a single genomic DNA extraction was performed from 200 µL of EDTA-stabilised sample using the Promega AX9760 protocol (Promega Corporation^TM^, Madison, WI, USA) for whole blood. The process was automated using the KingFisher^TM^ Flex Purification System (Thermo Fisher Scientific^TM^, Waltham, MA, USA), with all reagents handled at room temperature (15–30 °C) and according to the manufacturer’s specified conditions.

The extraction workflow was conducted according to the manufacturer’s instructions. An internal extraction control (IEC) was added to each sample to monitor the efficiency and integrity of the process.

Extracted DNA was not quantified prior to real-time PCR, and no inter-sample normalisation (e.g., to total DNA mass or a host reference gene) was performed, because the study was designed to determine PCR positivity rather than to infer bacterial load. Instead, a fixed input of 5 µL of extracted DNA was used per reaction for all samples, thereby standardising the reaction template volume while the IEC monitored extraction performance.

Subsequent molecular detection of *B. henselae* DNA was performed using the NZYtech *Bartonella henselae* qPCR Kit^®^ (NZYtech^TM^, Lisbon, Portugal). Real-time PCR detection of *B. henselae* DNA was performed using the NZYtech *B. hensela*e qPCR Kit, a TaqMan^®^ assay with *B. henselae*-specific primers targeting the *gltA* gene [73]. The manufacturer reports >95% homology across a broad range of *B. henselae* genomes and no detection of other closely related species. Under optimal conditions, the kit shows priming efficiency of >95% and a reported detection limit of at least 10 target copies per reaction.

Real-time qPCR was chosen due to its recognised status as a highly specific reference method for detecting *B. henselae* DNA [18,74,75,76,77,78], providing robust analytical performance in routine molecular screening.

PCR reactions were assembled with 15 µL of master mix and 5 µL of extracted DNA per well, including positive and negative controls. Reactions were loaded into a 96-well plate, sealed with optical adhesive film, centrifuged briefly to eliminate air bubbles, and processed on a QuantStudio^TM^ 5 Real-Time PCR System (Thermo Fisher Scientific^TM^, Waltham, MA, USA) following the thermal cycling conditions suggested in the manufacturer’s protocol (Table 1).

Each qPCR run incorporated a positive control (the kit-supplied *B. henselae* DNA standard of known concentration) and a non-template negative control (NTC; nuclease-free water) to verify assay performance.

Fluorescence was monitored in real time through a fluorogenic probe system comprising a 5′ reporter dye and 3′ quencher. During the extension phase, probe cleavage by Taq polymerase resulted in increased fluorescence, directly proportional to the amount of amplified target.

For the final validation of the results, the following criteria had to be met:

- Amplification of the internal extraction control;
- No amplification of the negative control;
- Amplification of the positive control.

Only results meeting these criteria were validated as negative (no amplification of the target), positive (true amplification of the target, indicated by a sigmoidal curve), or invalid (if there was no amplification of the target and no amplification of the internal extraction control, and therefore excluded from the study).

Data interpretation and analysis were performed using the Design & Analysis 2 (DA2) software, version 2.8 (Thermo Fisher Scientific^TM^, Waltham, MA, USA), in accordance with the manufacturer’s recommendations. The software was employed to extract both qualitative and quantitative parameters, including Ct values and amplification curve profiles, based on predefined analytical settings provided by the manufacturer. Ct values were used solely for qualitative confirmation of valid target amplification, and no quantitative interpretation or statistical comparison of Ct values was performed.

### 2.3. Confirmatory Nested PCR and Sanger Sequencing

To confirm the real-time PCR findings, five randomly selected blood specimens (three qPCR-positive and two qPCR-negative) were submitted to the reference Centre for the Study of Vectors and Infectious Diseases, Dr. Francisco Cambournac (CEVDI), Águas de Moura, Portugal. Genomic DNA was re-extracted from 200 µL of EDTA-anticoagulated whole blood using MagCore^®^ Automated Extraction Kits (RBC Bioscience Corp., New Taipei City, Taiwan), according to the manufacturer’s instructions. Samples were then analysed by conventional nested PCR targeting a partial fragment of the *ribC* gene (riboflavin synthase), as previously described [79]. Amplicons from positive specimens were purified with ExoSAP-IT^®^ (Affymetrix, Santa Clara, CA, USA) following the manufacturer’s protocol and sequenced bidirectionally by the Sanger method. Chromatograms were manually inspected, and sequences were aligned and trimmed using the BioEdit Sequence Alignment Editor v7.7.1.0 (Tom Hall, Ibis Biosciences, Carlsbad, CA, USA). Final sequences were compared with reference sequences available at GenBank (NCBI) using BLASTn (https://blast.ncbi.nlm.nih.gov/Blast.cgi, accessed on 29 December 2025).

### 2.4. Statistical Analysis

Data were extracted from Sislab^®^ (Glintt, Global Intelligent Technologies, Lisbon, Portugal) and organised in Microsoft Excel^®^ (Microsoft, Redmond, WA, USA). Data integrity and completeness were verified prior to analysis. All statistical procedures were conducted using JMP^®^ v18.0.0 (SAS Institute, Cary, NC, USA) and DATAtab^®^ (numiqo e.U., Graz, Austria, 2025).

Descriptive statistics were computed for all variables. Data normality was assessed with the Shapiro–Wilk test, and non-parametric methods were applied as assumptions of normality were not met (*p* < 0.05).

Associations with categorical variables (NUTS 2 region, sex and breed) and PCR result (negative/positive) were evaluated using Fisher’s exact test when more than 20% of expected cell frequencies were below five; otherwise, Pearson’s chi-squared test was applied. Pearson’s contingency coefficient (C) was calculated to quantify the effect size. For continuous variables, such as age (years), differences between PCR-positive and PCR-negative cats were assessed using the Mann–Whitney U test.

Univariable logistic regression analyses were then performed to estimate odds ratios (OR) and 95% confidence intervals (CI) for potential predictors of *B. henselae* DNA positivity. Variables with *p* < 0.10 were subsequently entered into a multivariable logistic regression model. A *p*-value ≤ 0.05 was considered statistically significant [80,81].

## 3. Results

### 3.1. General Positivity and Geographical Location

Of the total of 270 cats tested in this study, 47 (17.4%; 95% CI: 13.1–22.5%) were positive for *B. henselae*, while the remaining 223 (82.6%; 95% CI: 77.5–86.9%) yielded negative results. Three positive samples independently processed at CEVDI confirmed the identification of *B. henselae* by sequence analysis. All sequences were identical, and BLAST results showed 100% nucleotide identity (223/223 bp) with *B. henselae* strain Houston-I (GenBank accession number: CP020742.1).

Geographical location (NUTS 2) was available for all 270 cats. Most submissions originated from the North (*n* = 242, 89.6%), followed by the Centre (*n* = 18, 6.7%) regions. Smaller contributions were recorded for the Autonomous Region of Madeira (*n* = 3, 1.1%), the Autonomous Region of the Azores (*n* = 3, 1.1%), the Algarve (*n* = 2, 0.7%), Lisbon (*n* = 1, 0.4%) and Setúbal Peninsula (*n* = 1, 0.4%). No data were available for any other geographical regions.

PCR positivity was confined to three NUTS 2 regions, namely the North (*n* = 42), the Centre (*n* = 3) and the Autonomous Region of the Azores (*n* = 2). No PCR-positive cats were identified in any other regions. Despite this distribution, there was no statistically significant association between the NUTS 2 region and *B. henselae* DNA positivity (Fisher’s exact test, *p* = 0.478). To improve interpretability, regions with fewer than five observations were subsequently grouped into three categories: North, Centre, and Other regions. Yet the association remained non-significant (Fisher’s exact test, *p* = 0.921). Regional comparisons were limited by sampling imbalance, with most submissions originating from the North. Consequently, the absence of positives in some regions is more plausibly attributable to small sample sizes than to true absence of infection.

### 3.2. Age

From the 270 cats analysed for *B. henselae* DNA, age data were available for 200 individuals; for the remaining 70 cats (25.9%), age was not specified in the requisition file, and these were therefore excluded from age-related analyses. Age distribution among these 200 cats ranged from 0.3 years (≤1 year) to 15 years (≥11 years), with a median age of 3 years (interquartile range [IQR] 1–5 years). PCR-positive cats were significantly younger than PCR-negative cats (median age 2 years [IQR 1–5] vs. 3 years [IQR 1–6]; Mann–Whitney U = 2241.5, Z = −2.08, *p* = 0.037). Table 2 displays *B. henselae* DNA positivity according to age group.

### 3.3. Sex

Of the 270 cats analysed, 144 (53.3%) were females and 126 (46.7%) were males. Among females, 20 (13.9%, 95% CI 9.2–20.5) tested positive, whereas 27 (21.4%, 95% CI 15.2–29.4) of the males were positive. Although infection was more frequent in males, the difference between sexes was not statistically significant (χ^2^ = 2.69, *df* = 1, *p* = 0.103; C = 0.14).

### 3.4. Breed

European Shorthair cats predominated in the cohort (*n* = 254, 94.1%, 95% CI 90.6–96.3), followed by Persian (*n* = 6, 2.2%), Scottish Fold (*n* = 3, 1.1%), Siamese (*n* = 2, 0.7%), Scottish Straight (*n* = 2, 0.7%), Sphynx (*n* = 2, 0.7%) and Maine Coon (*n* = 1, 0.4%). All PCR-positive cats were European Shorthair (*n* = 47, 17.4%, 95% CI 13.4–22.4), with no positive results recorded in any of the remaining breeds. Although PCR positivity was observed exclusively in European Shorthair cats, no statistically significant association was found between breed and *B. henselae* DNA positivity (Fisher’s exact test, *p* = 0.730). After grouping breeds with sparse counts into broader categories, the result remained non-significant (*p* > 0.05).

### 3.5. Univariable and Multivariable Logistic Regression

Univariable logistic regression identified age as the only variable significantly associated with *B. henselae* DNA positivity. Each additional year of age decreased the odds of infection by approximately 17% (OR = 0.83, 95% CI 0.70–0.98, *p* = 0.0125). Sex (*p* = 0.105) and NUTS 2 region (*p* = 0.947) were not significant predictors.

The multivariable logistic regression model was not performed as age was the only predictor of *B. henselae* infection. Table 3 displays univariable logistic regression for predictors of *B. henselae* infection.

## 4. Discussion

In this study, *B. henselae* DNA was detected in 17.4% of tested cats, which is broadly consistent with the epidemiological range reported in similar climates [1,35,36,37,48,52,82,83,84]. Previous surveys in Portugal revealed marked variability: a large southern study reported only 2.9% PCR-positive cats [30], whereas an urban Lisbon survey of stray cats reported about 20% positivity [1]. Our finding falls between these extremes and closely aligns with the pooled Western European prevalence (~19%) and the global average (~15%) [35], suggesting that *B. henselae* circulates in Portuguese cats at levels comparable to other temperate European contexts. Importantly, even within Europe, *B. henselae* prevalence can vary widely—from as low as 2.9% in some areas of Portugal up to ~40% in certain Polish studies—a circumstance that underscores the influence of local ecology, host population and sampling factors [40,41,63].

Any apparent geographical clustering in our dataset should be interpreted cautiously. Submissions were highly unbalanced across NUTS 2 regions (North: 242/270, 89.6%), and PCR positivity being confined to the North, Centre and Autonomous Region of the Azores occurred despite no statistically significant association with region (*p* = 0.478; after grouping into three categories—North, Centre and Other regions—*p* = 0.921), which strongly suggests limited power to detect true spatial differences rather than genuine absence elsewhere. Warmer and humid climates generally favour year-round flea survival and higher cat infection prevalences [34,85,86,87,88,89]. In Portugal, the overall mild climate may permit sustained flea activity nationwide. Therefore, any apparent geographical heterogeneity in feline *B. henselae* is more plausibly explained by local vector pressure than by a simple “indoor vs. outdoor” dichotomy. Humid coastal microclimates can support persistent flea populations, and high urban cat density (multi-cat households and nearby colonies) may facilitate flea exchange. Differences in owner practices and access to veterinary care may further translate into variable consistency of ectoparasiticide use. Collectively, these factors provide a coherent explanation for higher urban prevalences (e.g., Lisbon city) even among predominantly owned, indoor cats, while lower observed PCR-positivity may occur in less densely populated settings where flea exposure is reduced and prevention is applied more consistently [35,40,44,89,90,91,92,93].

This spatial heterogeneity is clinically relevant for feline blood banks. *Bartonella* spp. infection is frequently subclinical and can be associated with prolonged bacteraemia, meaning apparently healthy donor cats, particularly from urban/coastal areas with higher flea pressure, may still harbour transfusion-transmissible infection. Donor programmes should accordingly prioritise strict year-round ectoparasite control and include *Bartonella* spp. PCR within routine screening panels, as serology alone cannot reliably exclude occult infection [13,27,40,94,95,96].

In terms of risk factors, our findings are consistent with international reports, with younger age being associated with PCR positivity. Each additional year of age reduced infection odds by approximately 17%, consistent with reports identifying juvenile cats (<2 years) as a key risk group, likely reflecting greater flea exposure and outdoor activity [30,44,45,46,47]. No associations were observed for sex, although infection was slightly more frequent in males (21% vs. 14%); this difference was not statistically significant, consistent with the lack of a reproducible sex predisposition across most studies [44,45,46,48,49,50]. Likewise, all infected cats in our study were European Shorthair, the predominant breed in the present sample, and no breed effect was evident, in agreement with the literature indicating no consistent breed association [40,48,90]. Taken together, these findings support the prevailing view that *Bartonella* spp. infection risk in cats is primarily driven by environmental exposure (flea infestation, outdoor access, and dense cat populations) rather than intrinsic host characteristics [35,40,44,64,90,91,92,93,94].

### 4.1. Public Health and One Health Implications

From a One Health perspective, the detection of *B. henselae* in apparently healthy cats has implications for both veterinary and human health. Infected cats usually remain carriers, and clinical illness in cats (e.g., endocarditis) is rare in Portugal, as in other Southern European settings [44]. However, cats serve as the reservoir for this zoonosis, and bacteraemic cats can transmit the organism to people through flea dirt that contaminates scratches or bites [27,92,97,98]. CSD in humans is generally an innocuous, self-limiting infection causing regional lymphadenopathy and fever, but it can have atypical manifestations and lead to serious complications (such as neurobartonellosis, bacillary angiomatosis or endocarditis), especially in immunocompromised individuals [27,42,96,97,98,99,100,101,102,103,104,105,106]. Indeed, the potential for severe outcomes—albeit infrequent—means that the public health risk cannot be ignored. Portugal has only sporadic official reports of human CSD and related syndromes, like other countries where feline *Bartonella* spp. are endemic [1,42,43]. This situation likely reflects the fact that while many cats harbour *B. henselae*, transmission to humans requires specific circumstances (e.g., a scratch from a flea-infested cat) and most infections in healthy people are resolved without diagnosis. Veterinary and public health professionals should collaborate to mitigate the risk of *Bartonella* spp. spread. Rigorous flea control in cats is paramount, as cat fleas (*C. felis*) are the principal vectors maintaining the bacterium in cat populations and indirectly transmitting it to humans. Nonetheless, our findings reinforce the need for vigilance and preventive measures to reduce zoonotic transmission.

### 4.2. Methodological Limitations and Diagnostic Considerations

In the present study, prevalence should be interpreted conservatively. We used a single EDTA-blood, species-specific (*B. henselae*) qPCR, which, despite high analytical specificity, has limited sensitivity for *Bartonella* spp. because feline infection is often occult and characterised by intermittent, low-grade bacteraemia. Infected cats may test PCR-negative at a given time point, particularly after recent antimicrobial exposure. In addition, a *B. henselae-*only assay will miss other feline *Bartonella* spp., such as *B. clarridgeiae*, which likely leads to underestimation of the overall *Bartonella* spp. burden. We did not perform culture or enrichment culture, so we could not confirm viable infection or undertake strain typing. We also did not include serology, which may capture prior exposure but cannot distinguish active bacteraemia. These constraints collectively favour under-ascertainment. Importantly, a negative blood PCR does not exclude infection at the individual level.

The study population consisted of apparently healthy cats tested as part of preventive vector-borne disease screening, which may still limit the representativeness of the broader feline population. Submissions were geographically skewed towards Northern Portugal, which reduced the ability to compare regions. Key exposure variables such as flea infestation, ectoparasite prevention, and outdoor access were not recorded.

### 4.3. Future Directions

Future work should adopt regionally representative sampling across owned, shelter, and free-roaming cats, with standardised recording of key exposure variables (e.g., flea infestation status, ectoparasite prevention practices, outdoor access, and household density). Methodological refinement should prioritise broader molecular assays (genus-level and/or multiplex), ideally complemented by enrichment culture to improve detection and enable isolate recovery. In parallel, focused evaluation of feline reproductive tissues may help assess the plausibility of transplacental exposure and clarify potential reproductive implications. Finally, One Health integration would be strengthened through dedicated surveys in high-risk human groups and further optimisation of feline blood bank protocols, including routine PCR screening for *Bartonella* spp. in donors and strict year-round ectoparasite control.

## 5. Conclusions

The present study provides molecular evidence that *B. henselae* is endemic in Portuguese cats, with a 17.4% prevalence consistent with reports from other temperate, flea-permissive European regions. Despite the North-biased, laboratory-submitted sampling frame and the use of a single-timepoint *B. henselae*-specific qPCR on EDTA blood, true prevalence was probably underestimated due to intermittent bacteraemia and undetected non-*B. henselae* species. Even so, these findings indicate sustained, subclinical circulation within the feline population. Younger age was the only independent predictor of infection, supporting an exposure-driven model in which infection risk is primarily shaped by flea pressure and preventive management rather than region, sex, breed, or intrinsic host factors.

Clinically, the identification of *B. henselae* DNA in clinically healthy cats has direct relevance for feline blood banks and transfusion medicine, as apparently healthy donors may remain bacteraemic for prolonged periods. Blood bank protocols should, therefore, prioritise strict year-round flea control and include PCR for *Bartonella* spp. in donor routine screenings, recognising that serology alone cannot reliably exclude occult infection. Future work should expand regionally balanced sampling across owned, shelter and stray cats, adopt broader molecular targets for multiple feline *Bartonella* spp., and link infection status to quantified vector exposure and management practices to refine risk estimates and strengthen One Health prevention at the cat–flea–human interface.

## Figures and Tables

**Table 1:** Thermal cycling conditions used for the detection of *Bartonella henselae* in EDTA-anticoagulated whole blood suggested in the manufacturer’s protocol.

Cycles	Temperature	Time	Notes
1	95 °C	2 min	Polymerase activation
40	95 °C	5 s	Denaturation
	60 °C	30 s	Annealing/Extension

**Table 2:** Distribution of PCR results for *Bartonella henselae* (DNA) among cats by age group.

		*Bartonella henselae* (DNA)					
	Negative			Positive			Total
Age Groups	*n*	% Within Age Groups	95% CI	*n*	% Within Age Groups	95% CI	*n*
<1 year	19	79.2%	59.5–90.8	5	20.8%	9.2–40.5	24
1 to <2 years	26	78.8%	62.2–89.3	7	21.2%	10.7–37.8	33
2 to <6 years	81	81.8%	73.1–88.2	18	18.2%	11.8–26.9	99
6 to <11 years	30	85.7%	70.6–93.7	5	14.3%	6.3–29.4	35
≥11 years	9	100%	70.1–100.0	0	0.0%	0.0–29.9	9
Total	165	82.5%	-	35	17.5%	-	200

CI, confidence interval. Age grouping was retained for descriptive purposes only. Statistical testing was based on the continuous variable.

**Table 3:** Univariable logistic regression for predictors of *Bartonella henselae* infection.

Variable	Category/Unit	Univariable OR (95% CI)	Wald χ^2^	*p*-Value
Age (years)	Continuous	0.83 (0.70–0.98)	6.23	0.013
Sex	Male vs. Female (*)	1.65 (0.90–3.23)	2.64	0.105
Region (NUTS 2)	Others vs. North (*)	1.03 (0.33–2.68)	0.004	0.947

CI, confidence interval; OR, odds ratio; NUTS 2, Nomenclature of Territorial Units for Statistics level 2 region. (*) Categorical predictors were modelled with the following reference categories: female for sex, and North for region (NUTS 2).

## Data Availability

The data presented in this study are available upon request from the corresponding authors.

## Abbreviations

The following abbreviations are used in this manuscript:

BAPGM	Bartonella alpha-proteobacteria growth medium
CEVDI	Centre for the Study of Vectors and Infectious Diseases Dr. Francisco Cambournac
CI	confidence interval
CSD	cat-scratch disease
Ct	cycle threshold
DA2	Design and Analysis 2 (software)
DNA	deoxyribonucleic acid
EDTA	ethylenediaminetetraacetic acid
IEC	internal extraction control
IHMT	Institute of Hygiene and Tropical Medicine
INSA	National Institute of Health
IQR	interquartile range
NTC	non-template control
NUTS 2	Nomenclature of Territorial Units for Statistics, level 2
PCR	polymerase chain reaction
qPCR	quantitative PCR
